# Supplementary material for: Saccharomyces boulardii (CNCM I-745) improves intestinal damage in sepsis by remodeling intestinal flora structure
Source: Front Cell Infect Microbiol. 2025 Jul 31;15:1602792. doi: 10.3389/fcimb.2025.1602792 (PMC12350329; doi:10.3389/fcimb.2025.1602792)
Supplement: Supplementary file 1 [file DataSheet1.zip › summary/summary/7_Differential_analysis/2_LEfSe_result/Group_DW4_vs_CW4_vs_BW4_vs_AW4_0.05_3.0/LEfSe_Group_DW4_vs_CW4_vs_BW4_vs_AW4.cladogram.pdf]

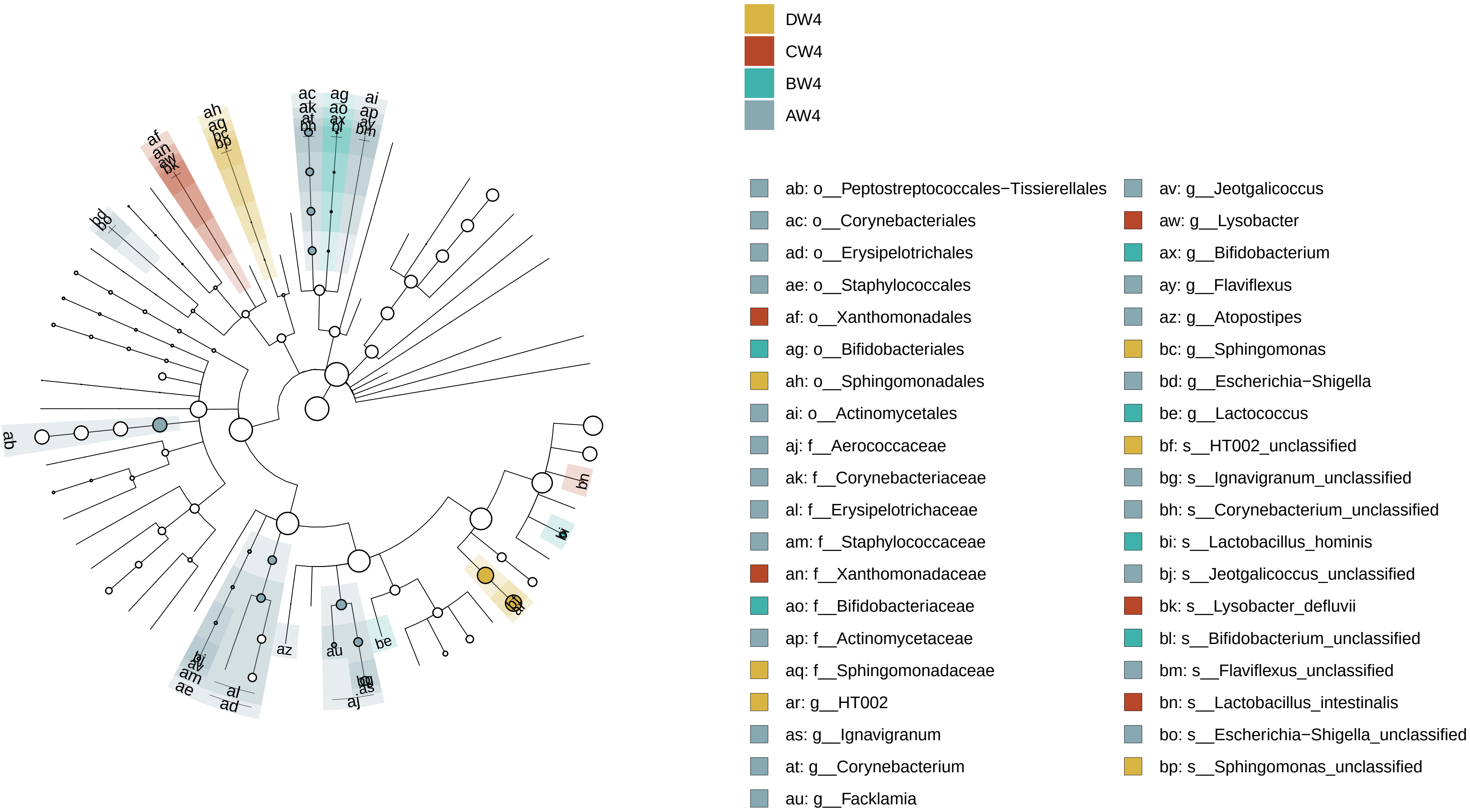

DW4
CW4
BW4
AW4
ab: o__Peptostreptococcales−Tissierellales
ac: o__Corynebacteriales
ad: o__Erysipelotrichales
ae: o__Staphylococcales
af: o__Xanthomonadales
ag: o__Bifidobacteriales
ah: o__Sphingomonadales
ai: o__Actinomycetales
aj: f__Aerococcaceae
ak: f__Corynebacteriaceae
al: f__Erysipelotrichaceae
am: f__Staphylococcaceae
an: f__Xanthomonadaceae
ao: f__Bifidobacteriaceae
ap: f__Actinomycetaceae
aq: f__Sphingomonadaceae
ar: g__HT002
as: g__Ignavigranum
at: g__Corynebacterium
au: g__Facklamia
av: g__Jeotgalicoccus
aw: g__Lysobacter
ax: g__Bifidobacterium
ay: g__Flaviflexus
az: g__Atopostipes
bc: g__Sphingomonas
bd: g__Escherichia−Shigella
be: g__Lactococcus
bf: s__HT002_unclassified
bg: s__Ignavigranum_unclassified
bh: s__Corynebacterium_unclassified
bi: s__Lactobacillus_hominis
bj: s__Jeotgalicoccus_unclassified
bk: s__Lysobacter_defluvii
bl: s__Bifidobacterium_unclassified
bm: s__Flaviflexus_unclassified
bn: s__Lactobacillus_intestinalis
bo: s__Escherichia−Shigella_unclassified
bp: s__Sphingomonas_unclassified
